# Assessing Telemetry Utilization in Inpatient Care: Establishing and Evaluating the Impact of a Telemetry Admission Protocol

**DOI:** 10.7759/cureus.113419

**Published:** 2026-07-26

**Authors:** Miguel Santos Lopez, Luis Colon Gonzalez, Gabriela L Lacourt Sosa, Yomara Huertas Gomez, Vanessa Rodriguez Mier, Alondra Cruz Rodríguez, Dargee Milibeth Torres Guevárez, Gisela Feliciano

**Affiliations:** 1 Internal Medicine, Centro Médico Episcopal San Lucas, Ponce, PRI; 2 Cardiology, Centro Médico Episcopal San Lucas, Ponce, PRI; 3 Medicine, Ponce Health Sciences University, Ponce, PRI; 4 Medicine, Universidad Central del Caribe, Bayamón, PRI; 5 Sciences, Pontificia Universidad Católica de Puerto Rico, Ponce, PRI

**Keywords:** cardiology devices, cardiology research, telemetry monitoring, telemetry overuse, telemetry utilization

## Abstract

Cardiac telemetry monitoring is an essential tool for assessing cardiac function in hospitalized patients; however, its overuse in low-risk populations can increase healthcare costs, alarm fatigue, and unnecessary resource utilization. This study aimed to assess the use of cardiac telemetry at a tertiary care center in Puerto Rico. Using a retrospective cross-sectional review of medical records, we evaluated whether telemetry was used appropriately according to established clinical criteria and identified patterns of overuse. The findings provide a foundation for developing an institutional telemetry protocol to optimize telemetry utilization, enhance patient safety, and improve cost-effectiveness. A total of 822 hospitalized patients receiving cardiac telemetry were included. Variables such as history of cardiac disease, comorbidities, length of stay, indication for telemetry, and duration of monitoring were analyzed. Our findings revealed that 476 (57.9%) patients received telemetry for non-evidence-based reasons, while 346 (42.1%) had clear clinical indications. Patients with non-evidence-based telemetry had longer monitoring durations, averaging 5.68 ± 5.90 days compared to 2.98 ± 2.94 days for those with evidence-based indications. They also spent slightly less time in the emergency department, with a mean of 7.18 ± 4.73 hours, compared to 7.98 ± 6.44 hours for the evidence-based group. Although not statistically significant (p = 0.137), the mean length of stay was higher among non-evidence-based cases (6.73 ± 8.25 days) than among evidence-based cases (4.46 ± 7.38 days). These differences contributed to an estimated cost of $4,711 per patient for non-evidence-based telemetry compared to $3,122 per patient for evidence-based monitoring. This study suggests that implementing standardized, evidence-based guidelines for telemetry initiation and discontinuation could reduce overuse, lower costs, decrease alarm fatigue, and maintain patient safety while showcasing the potential benefits of a hospital-wide protocol to optimize telemetry practices.

## Introduction

Cardiac telemetry is an important tool for inpatient monitoring of cardiac events. Its use enables the detection of life-threatening arrhythmias, thereby improving patient management and clinical outcomes [1]. Despite its benefits, overuse of telemetry is common in hospitals that lack a standardized protocol for its implementation. Excessive utilization can lead to increased healthcare costs, unnecessary use of hospital resources, and alarm fatigue without providing additional clinical benefit [2]. Despite the widespread use of cardiac telemetry, evidence-based data evaluating appropriate utilization criteria within Puerto Rico remains limited. At this tertiary care center, the initiation and discontinuation of telemetry monitoring are not currently guided by a standardized institutional protocol, resulting in variability in its application and duration of use. As a result, it is unclear whether telemetry is being applied in accordance with evidence-based guidelines or whether its use exceeds appropriate limits, thereby contributing to increased costs and misallocation of resources.

The American Heart Association (AHA) provides evidence-based recommendations for the use of telemetry monitoring. These guidelines are designed to ensure effective use of telemetry [3]. However, data on adherence to these guidelines in minority populations, such as those in Puerto Rico, remain limited. This lack of data highlights gaps in our understanding of the appropriateness of telemetry utilization in these populations and its impact on healthcare resources.

Previous studies have shown that the inappropriate use of cardiac telemetry is a growing phenomenon, with up to 43% of patients receiving telemetry without evidence-based indications [4]. The financial burden of telemetry is estimated to be substantial. In a study by Chong-Yik et al. [5], the cost of telemetry in a tertiary care hospital in the United States was estimated to be approximately $34.28 USD per patient-day higher than that of a hospital stay without telemetry. The study further found that eliminating inappropriate telemetry utilization could yield annual savings of approximately $528,241 USD. Beyond the financial burden, overuse of telemetry can also affect clinical effectiveness. Excessive monitoring may lead to desensitization among clinicians, reducing their responsiveness to alarms and potentially diminishing the sensitivity of telemetry to detect clinically significant events. Studies have shown that between 80% and 99% of telemetry alarms in clinical and intermediate care units were either false or clinically insignificant, leading to severe alarm fatigue [6].

The primary objective of this study was to assess the appropriateness of cardiac telemetry utilization among hospitalized patients at a tertiary care center in Puerto Rico by determining the proportion of telemetry orders that aligned with established evidence-based clinical criteria.

Secondary objectives included evaluating differences in telemetry monitoring duration, hospital length of stay, emergency department time, mortality outcomes, and estimated costs between patients receiving evidence-based and non-evidence-based telemetry. Additionally, this study aimed to identify patterns of telemetry utilization across clinical specialties to inform future quality improvement initiatives and the development of institutional telemetry management protocols.

## Materials and methods

This study is a retrospective cross-sectional record review conducted at a tertiary care center in Puerto Rico. The study included patients aged 21 years or older who received cardiac telemetry monitoring during a single admission between January 1 and December 31, 2024.

Patient data were obtained from the hospital's electronic medical record system and stored in the institutional REDCap, a HIPAA-compliant data storage system [7]. Inclusion criteria were patients who underwent telemetry monitoring. Exclusion criteria included patients younger than 21 years and those with incomplete medical records. Variables were collected using structured REDCap surveys that included multiple-choice and single-choice responses, a text box for continuous variables, and date fields for admission and discharge dates to calculate length of stay. Variables included demographic characteristics (age, sex); relevant medical history (cardiac disease); comorbidities (e.g., hypertension, diabetes, chronic kidney disease); reason for hospital admission; indication for telemetry; duration of telemetry monitoring; and length of hospital stay.

The appropriateness of telemetry was assessed in accordance with AHA guidelines, which define evidence-based indications for initiating and discontinuing continuous cardiac monitoring [8]. Patients were classified into two groups based on a dichotomous variable: those receiving telemetry for evidence-based indications and those receiving telemetry without clear evidence-based indications. Evidence-based indications were based on Class I (syncope with complications, second- or third-degree AV block, atrial fibrillation, post-operation cardiac complications, initiation of antiarrhythmic medications, drug toxicity with arrhythmia, and external pacemakers); Class II (patients with acute coronary syndrome, chronic heart failure, acute myocardial infarction, chest pain, unstable angina, syncope with normal condition, post-operative patients with a history of cardiac intervention, symptomatic bradycardia, cardiac confusion, stroke, myocarditis or pericarditis, electrolyte disturbances, and cardiac or respiratory arrest); and Class III (post-cardiac invasive procedures: pacemaker placement, ablation, angiography, etc.). This information was obtained by reviewing patients' order histories to determine whether they were evidence-based or non-evidence-based. Investigators classified each patient according to the applicable AHA telemetry indication. The principal investigator then revised and confirmed the information.

Telemetry cost estimates were obtained from internal financial data and rounded to an estimated standardized value of $700 USD per patient-day for the analysis, as provided by the hospital's financial department. Rounding was used to avoid reporting precise institution-specific financial information and to provide a simplified cost value that can be generalized across similar healthcare settings in Puerto Rico. The site research coordinator conducted the analysis using the REDCap database.

Descriptive statistics were used to summarize demographic and clinical characteristics. Normal distribution analyses were performed to determine whether tests would be parametric or non-parametric. Comparisons between groups were performed using Student's t-tests or the Mann-Whitney U test, depending on the data distribution, to evaluate differences in telemetry monitoring duration, hospital length of stay, and estimated costs associated with telemetry utilization. Chi-square and Fisher's exact tests were used for dichotomous analyses. For descriptive analyses, continuous variables were reported as means ± standard deviations and categorical variables as frequencies and percentages. Statistical analyses were conducted using SPSS Statistics version 29.0.2.0.0.2.0 (IBM Corp. Released 2022. IBM SPSS Statistics for Windows, Version 29.0.2.0.0.2.0. Armonk, NY). Data were safely stored using REDCap. This study obtained Institutional Review Board approval from Ponce Health Sciences University under protocol number 2404192089.

## Results

**Figure 1:**
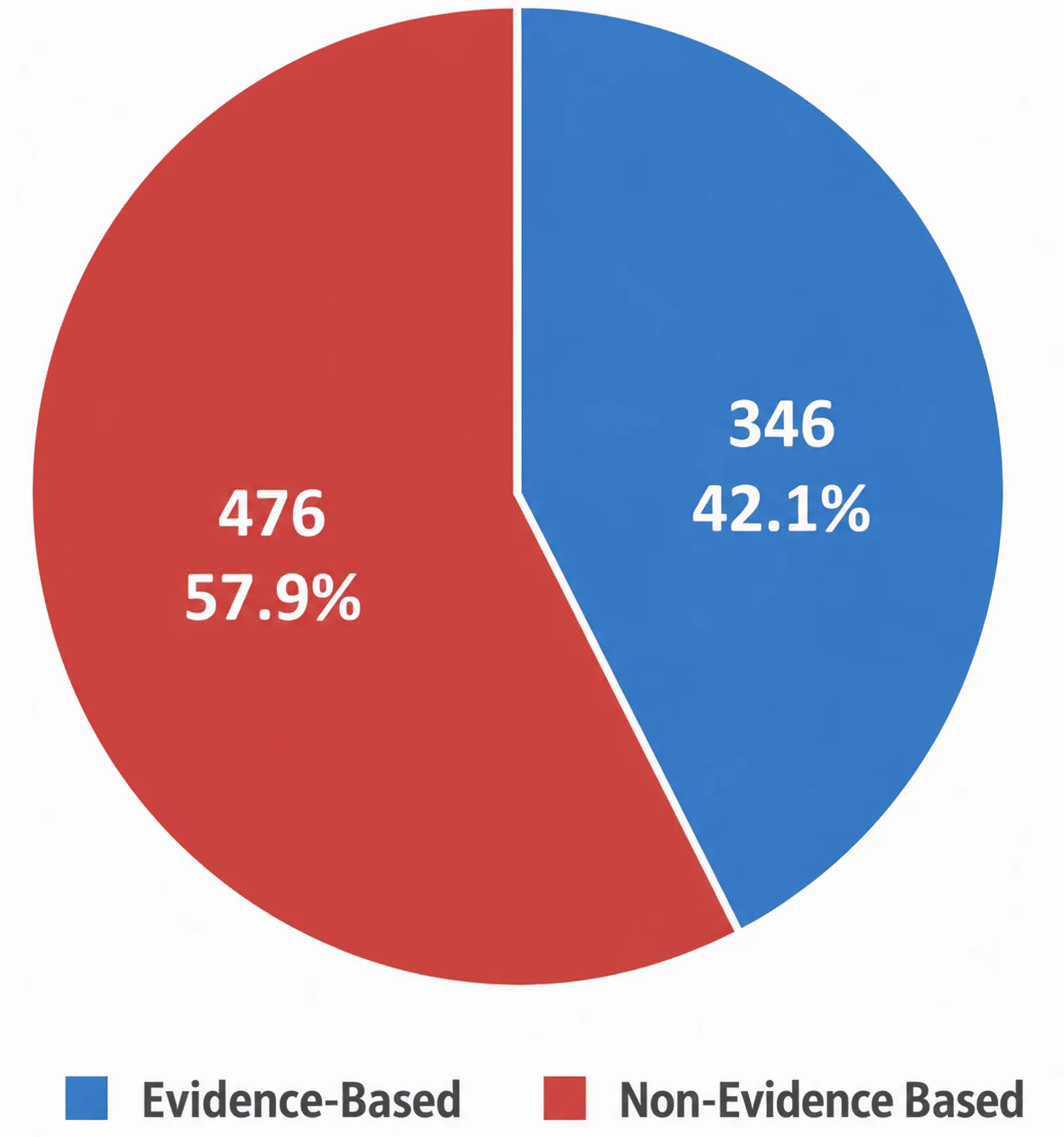
Graph comparing the percentages of evidence-based versus non-evidence-based use of telemetry based on the study population

A total of 822 telemetry orders were analyzed to assess adherence to evidence-based criteria and associated clinical outcomes. Overall, 476 (57.9%) orders were classified as non-evidence-based, reflecting substantial inappropriate utilization (Figure 1).

The study population consisted of 428 (52.1%) females and 394 (47.9%) males, with no statistically significant association between sex and telemetry appropriateness (p = 0.212; risk ratio = 1.194, 95% CI 0.91-1.58) (Table 1).

**Table 1:** Table comparing reason for telemetry with female and male demographics

			Reason for telemetry		
			Evidence-based	Non-evidence-based	Total
Sex	Female	Count	189	239	428
		Percentage %	54.6%	50.2%	52.1%
	Male	Count	157	237	394
		Percentage %	45.4%	49.8%	47.9%
Total		Count	346	476	822
		Percentage %	100.0%	100.0%	100.0%

Table 2 provides the comparative data for first-time telemetry admissions based on specialty. Internal medicine providers accounted for the majority of telemetry orders (613; 74.6%), followed by physician assistants (122; 14.8%), family medicine (38; 4.6%), and cardiology (18; 2.2%). Non-evidence-based telemetry was most prevalent in internal medicine (402 orders; 84.5%) and least prevalent in cardiology (four orders; 0.8%), suggesting variability in guideline adherence across specialties (Table 2).

**Table 2:** Table comparing reason for telemetry versus different subspecialties

		Reason for telemetry		
		Evidence-based	Non-evidence-based	Total
Specialty	Cardiology	14	4	18
		4.0%	0.8%	2.2%
	Family medicine	9	29	38
		2.6%	6.1%	4.6%
	Internal medicine	211	402	613
		61.0%	84.5%	74.6%
	Obstetrics and gynecology	1	0	1
		0.3%	0.0%	0.1%
	Other	3	6	9
		0.9%	1.3%	1.1%
	Physician assistant	102	20	122
		29.5%	4.2%	14.8%
	Surgery	0	8	8
		0.0%	1.7%	1.0%
	Specialties not documented	6	7	13
		1.7%	1.4%	1.6%
Total		346	476	822
		100.0%	100.0%	100.0%

Patients receiving non-evidence-based telemetry had longer monitoring durations, averaging 5.68 ± 5.90 days, compared with 2.98 ± 2.94 days for those whose telemetry utilization met established criteria. They also spent slightly less time in the ED, with a mean of 7.19 ± 4.73 hours, compared with 7.98 ± 6.44 hours for the evidence-based group. Although not statistically significant (p = 0.137), the mean length of stay was higher among non-evidence-based cases (6.73 ± 8.25 days) than among evidence-based cases (4.46 ± 7.38 days) (Table 3).

**Table 3:** Table comparing age, BMI, duration of telemetry, length of stay, and hours at ED versus reason for telemetry BMI: body mass index, ED: emergency department

	Reason for telemetry	N	Mean	Standard deviation	p-value
Age	Evidence-based	346	71.00	13.73	0.282
	Non-evidence-based	476	71.61	14.16	
BMI	Evidence-based	345	28.93	6.83	0.098
	Non-evidence-based	475	28.88	7.65	
Duration of telemetry	Evidence-based	346	2.98	2.94	<0.001
	Non-evidence-based	476	5.68	5.89	
Length of stay	Evidence-based	346	4.46	1.23	0.137
	Non-evidence-based	476	6.73	2.11	
Hours at ED	Evidence-based	346	7.98	6.44	<0.001
	Non-evidence-based	476	7.19	4.73	

**Figure 2:**
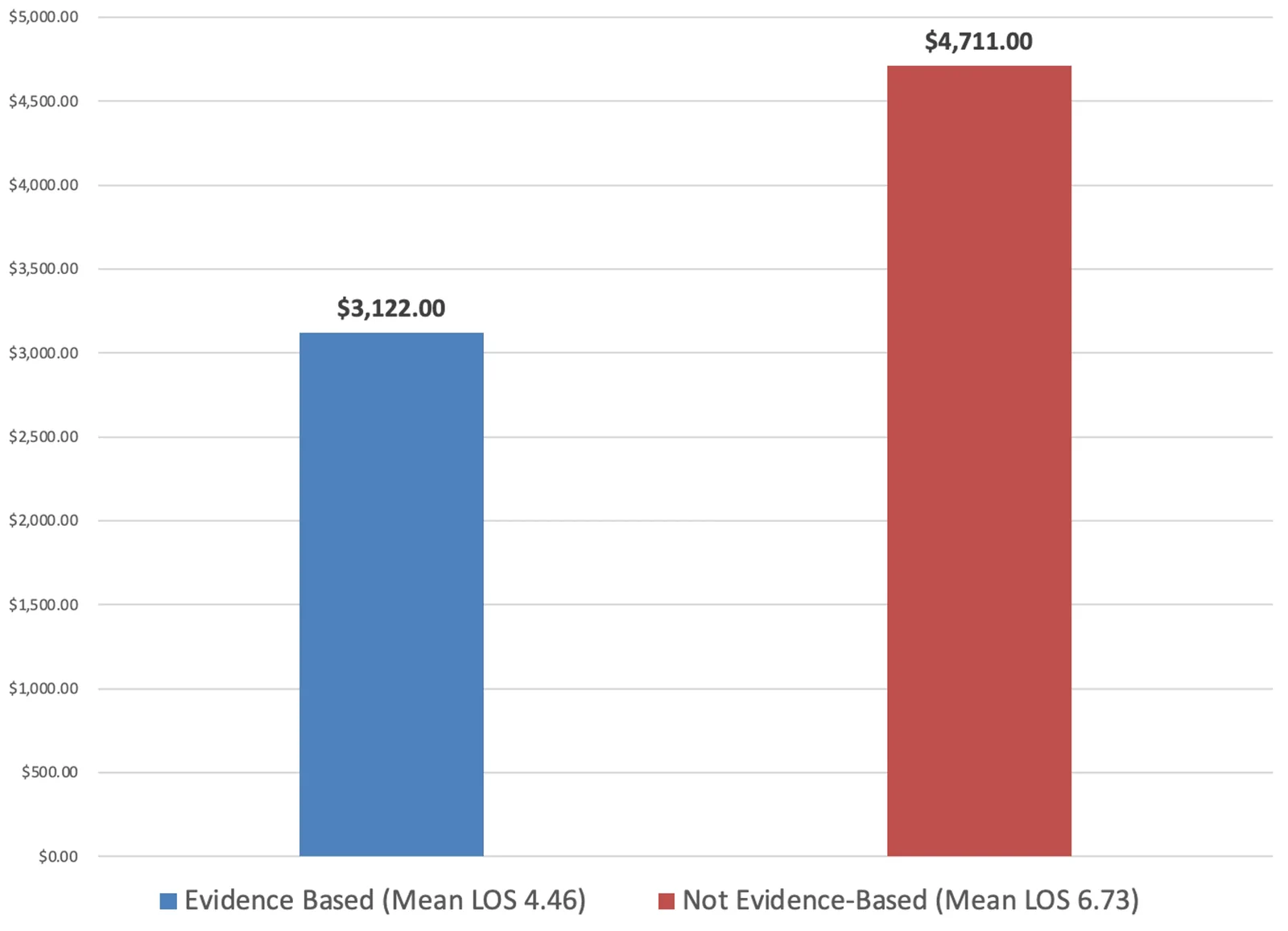
Cost analysis using an average $700.00 per patient-day for telemetry Cost per patient in USD

Estimated using an average cost of $700 USD for telemetry monitoring, this difference corresponds to an additional $1,589 USD per patient, with total mean costs of $4,711 USD for non-evidence-based cases and $3,122 USD for evidence-based cases (Figure 2).

Mortality analysis showed 39 in-hospital deaths (4.7%), with 23 deaths (59%) occurring among patients without evidence-based indications and 16 (41%) among those with appropriate telemetry utilization. This difference was not statistically significant (risk ratio = 1.10, 95% CI 0.545-2.013; p = 0.890) (Table 4).

**Table 4:** Table comparing reason for telemetry versus outcome of mortality in the study population

			Reason for telemetry		
			Correct	Not correct	Total
Death	No	Count	330	453	783
		% within death	42.1%	57.9%	100.0%
	Yes	Count	16	23	39
		% within death	41.0%	59.0%	100.0%
Total		Count	346	476	822
		% within death	42.1%	57.9%	100.0%
p = 0.890, risk 1.102 (0.545-2.013)					

## Discussion

Continuous telemetry monitoring is a critical tool for detecting life-threatening cardiac events, such as arrhythmias. Telemetry allows clinicians to manage high-risk patients more effectively. However, its overuse is detrimental to the analysis of patient outcomes. Its inappropriate use creates negative consequences like alarm fatigue, high costs, and extended hospitalizations [9]. Our results indicated that the majority of telemetry utilization was not evidence-based. This finding highlights persistent issues in inpatient care, particularly the use of cardiac monitoring in patients who do not meet established evidence-based criteria. Our findings suggest that telemetry overutilization may contribute to unnecessary healthcare expenditures and places an added burden on clinical staff. Moreover, excessive monitoring can lead to alarm fatigue and desensitization among healthcare workers, potentially compromising patient safety, response times, and clinical outcomes [3]. Our results are aligned with prior literature reporting high rates of inappropriate telemetry utilization in hospitals without standardized monitoring protocols. These findings emphasize the need for evidence-based criteria and provider education to guide telemetry ordering practices and promote more efficient resource utilization.

The proportion of inappropriate telemetry utilization observed in this study (57.9%) demonstrates a significant gap between current clinical practice and evidence-based recommendations. Although telemetry remains essential for patients at increased risk of arrhythmias and cardiac deterioration, our findings suggest that a considerable number of patients are being exposed to continuous monitoring without a clear indication. This pattern may reflect a broader challenge in inpatient medicine, where telemetry is often initiated as a precautionary measure and subsequently continued without reassessment of ongoing clinical need. The high proportion of non-evidence-based use observed in our institution is particularly relevant because it represents an opportunity for targeted quality improvement interventions within a healthcare system where standardized telemetry practices have not yet been established.

Beyond the frequency of inappropriate initiation, our results demonstrate that non-evidence-based telemetry was associated with substantially longer monitoring duration. Patients without evidence-based indications remained on telemetry for an average of 2.7 additional days compared with patients whose monitoring was clinically justified (5.68 ± 5.90 days vs. 2.98 ± 2.94 days). This difference suggests that the primary issue may not only be unnecessary telemetry initiation but also the failure to discontinue monitoring once the risk period has resolved. These findings highlight the importance of implementing both initiation and discontinuation criteria rather than focusing solely on appropriate ordering.

The specialty-specific distribution of telemetry utilization provides additional insight into potential areas for intervention. Internal Medicine services accounted for the majority of telemetry orders and represented the largest proportion of non-evidence-based utilization. This finding may be related to the complexity and heterogeneity of patients managed by internal medicine teams, who frequently care for patients with multiple comorbidities and uncertain clinical trajectories. However, the lower proportion of inappropriate telemetry utilization among cardiology services suggests that familiarity with cardiac monitoring guidelines may influence ordering practices. These results indicate that interventions targeting high-volume telemetry ordering services, particularly through education, clinical decision support, and standardized order requirements, may have the greatest impact. Although mortality was not significantly associated with evidence-based versus non-evidence-based telemetry utilization, this finding should be interpreted cautiously. The absence of a statistically significant difference does not demonstrate that unnecessary telemetry is harmless; rather, it suggests that inappropriate telemetry utilization did not demonstrate an observable mortality benefit within this study population. The primary consequences identified in this study relate to resource utilization, increased monitoring duration, and financial burden rather than direct mortality outcomes. Therefore, reducing unnecessary telemetry utilization should focus on improving healthcare efficiency while preserving monitoring for patients who are most likely to benefit.

The overuse of telemetry can be attributed to multiple factors, including clinician habits, fear of missing abnormal cardiac events, lack of awareness of guidelines, and system-related barriers [6]. Out of an abundance of caution, physicians may initiate cardiac monitoring when it's not necessary. Moreover, the absence of institutional policies or electronic order restrictions can prolong telemetry beyond the recommended duration. A previous study by Dressler et al. demonstrated substantial improvements in outcomes following implementation of revised telemetry orders. After its implementation, the mean weekly number of telemetry orders decreased from 1032.3 to 593.2.

Additionally, the mean telemetry usage duration was reduced from 57.8 hours to 30.9 hours [10]. In another study by Edholm et al. [11], telemetry utilization was reduced by 69% without an increase in mortality, code events, or care escalation following the 2015 implementation of a telemetry-reduction intervention that included education, process changes, routine feedback, and a financial incentive. Additionally, a study by Krouss et al. [12] demonstrated success in implementing an electronic health record (EHR) intervention that required mandatory documentation of indications for telemetry orders and a best-practice advisory (BPA) in an 11-hospital network in New York. The intervention led to a 19.5% reduction in average telemetry hours per patient encounter, with hospital-specific reductions ranging from 9% to 30%. The implemented BPA measure fired approximately 52,000 times. This resulted in the discontinuation of more than 27,900 telemetry orders.

Further supporting the finding that implementing system-wide measures is the most effective way to reduce telemetry overuse, a study by Chakravarthy et al. [13] demonstrated that education alone, though necessary, was insufficient for reducing unnecessary telemetry orders. As part of their quality improvement initiative, they evaluated the impact of a 15-minute educational session on appropriate telemetry indications over 78 weeks. Although Student's t-test indicated a statistically significant reduction in telemetry orders exceeding 48 hours (p = 0.002), segmented linear regression showed that this effect could not be attributed to the implemented intervention. Collectively, these results indicate that implementing system-wide interventions, such as EHR strategies, is the most effective way to substantially reduce the financial burden of telemetry overuse and improve patient outcomes.

Other studies have shown that limiting telemetry utilization to clinically necessary indications could significantly reduce in-hospital costs. Benjamin et al. suggested that limiting telemetry utilization could save at least $53 USD per patient per day. For a 400-bed hospital, an estimated $250,000 USD in annual cost could be saved [14]. Our findings further support the notion that reducing unnecessary telemetry utilization can substantially decrease the financial burden, highlighting that cardiac monitoring overuse contributes to significantly avoidable healthcare costs. In our study, assuming an average daily cost of $700 USD for telemetry, the estimated cost per patient without evidence-based indications was $4,711 USD, compared with $3,122 USD for those receiving evidence-based telemetry. These findings underscore the substantial financial burden associated with inappropriate telemetry utilization and highlight the potential cost savings achievable through adherence to evidence-based monitoring practices.

To address the overuse of telemetry, technology is evolving to make determining which patients require telemetry monitoring more seamless for physicians. Jaramillo et al. [15] demonstrated that the future of telemetry may involve an outpatient approach. A study conducted during the COVID-19 pandemic found that real-time at-home ECG monitoring was feasible due to technological advancements. Beyond financial considerations, telemetry is shifting toward a more advanced approach that leverages technologies such as artificial intelligence (AI). Lu et al. [16] noted that AI has been transforming cardiac telemetry from statistical models to advanced neural networks. Lastly, evidence suggests that remote patient monitoring systems, together with AI-powered wearable technology, could significantly reshape the future of telemetry while reducing healthcare utilization [17]. Combined with a more standardized system of telemetry implementation, these advancements could transform this vital, widely used tool into a more efficient system.

Study limitations

This study has several limitations. The retrospective design limits the ability to establish causal relationships, as findings are based on observed associations and hypothesis generation. Additionally, the single-center setting within Puerto Rico may limit the generalizability of the findings to other healthcare systems and patient populations. Potential misclassification bias may have occurred in the determination of evidence-based telemetry indications, given the reliance on available clinical documentation. Limitations inherent to medical record-based studies, including incomplete, inconsistent, or variable documentation, may have affected data accuracy. Recall bias may also have influenced the accuracy of collected information when clinical decisions or indications for telemetry utilization relied on retrospective provider documentation or recorded patient history. Furthermore, the absence of multivariable analytical methods limited the ability to adjust for potential confounding factors. Finally, case-mix bias should be considered when interpreting telemetry ordering patterns, as internal medicine services may be disproportionately represented due to their frequent role as primary consultants for telemetry management.

## Conclusions

Our findings suggest that a substantial proportion of telemetry utilization at our institution may not align with current evidence-based indications, highlighting an opportunity to optimize monitoring practices and promote more efficient use of healthcare resources. These findings support the need for institutional strategies aimed at improving adherence to evidence-based telemetry practices. Based on prior literature, interventions such as standardized criteria, provider education, and electronic decision-support tools may represent effective approaches to reduce inappropriate monitoring and improve resource utilization. Future studies should evaluate the impact of these interventions on telemetry utilization, patient safety outcomes, and healthcare costs.
